# Digital Assessment of Stained Breast Tissue Images for Comprehensive Tumor and Microenvironment Analysis

**DOI:** 10.3389/fbioe.2019.00246

**Published:** 2019-10-01

**Authors:** Shachi Mittal, Catalin Stoean, Andre Kajdacsy-Balla, Rohit Bhargava

**Affiliations:** ^1^Department of Bioengineering, Beckman Institute for Advanced Science and Technology, University of Illinois at Urbana–Champaign, Urbana, IL, United States; ^2^Department of Computer Science, University of Craiova, Craiova, Romania; ^3^Department of Pathology, University of Illinois at Chicago, Chicago, IL, United States; ^4^Departments of Mechanical Science and Engineering, Electrical and Computer Engineering, Chemical and Biomolecular Engineering, and Chemistry, Cancer Center at Illinois, University of Illinois at Urbana–Champaign, Urbana, IL, United States

**Keywords:** breast cancer, microenvironment, deep learning, ductal carcinoma *in-situ*, hyperplasia and clustering

## Abstract

Current histopathological diagnosis involves human expert interpretation of stained images for diagnosis. This process is prone to inter-observer variability, often leading to low concordance rates amongst pathologists across many types of tissues. Further, since structural features are mostly just defined for epithelial alterations during tumor progression, the use of associated stromal changes is limited. Here we sought to examine whether digital analysis of commonly used hematoxylin and eosin-stained images could provide precise and quantitative metrics of disease from both epithelial and stromal cells. We developed a convolutional neural network approach to identify epithelial breast cells from their microenvironment. Second, we analyzed the microenvironment to further observe different constituent cells using unsupervised clustering. Finally, we categorized breast cancer by the combined effects of stromal and epithelial inertia. Together, the work provides insight and evidence of cancer association for interpretable features from deep learning methods that provide new opportunities for comprehensive analysis of standard pathology images.

## Introduction

The current clinical pipeline for cancer diagnosis involves human expert evaluation of large sections of tissue stained with dyes or a variety of specialized molecular markers (Kiernan, 1999). Increasing cancer incidence rates put an increased burden on pathologists worldwide (Williams et al., 2017), as limited resources and limited growth of trained medical personnel (Robboy et al., 2015) are subjected to greater strains. In addition to the emerging needs for better diagnoses, challenges lie in accurate diagnoses using current methods as well (Robbins et al., 1995; Bahreini et al., 2015; Elmore et al., 2015; Stålhammar et al., 2016). Morphometric features form the bases of decisions today; however, difficulty in recognition of subtle morphologic changes and the process of assigning a discrete grade to a continuum of disease often makes the diagnosis prone to under or over-diagnosis. With the advent of whole slide imaging (Pantanowitz et al., 2011; Ghaznavi et al., 2013), digitized versions of stained slides are available and advanced computational analysis can be readily applied (Gurcan et al., 2009). Digital screening of images, even for simple classifications like “cancer” vs. “no cancer,” can help reduce the pathologist's workload (Veta et al., 2014) by triaging and focusing on specific areas as well as alerting them to borderline cases (Dong et al., 2014). Deep convolutional neural networks (CNN) are especially rising in popularity as the method of choice for image processing in medicine, due to their inherent ability to automatically extract features, ranging from those very general to those very specific to the problem under consideration (Ertosun and Rubin, 2015; Litjens et al., 2016; Bychkov et al., 2018; Coudray et al., 2018). Histopathology is an area where the CNN architectures can play an important role due to the intricacy of the decisions and the abundance of data resulting from routine patient screening, digital archiving of data and advances in imaging (Litjens et al., 2016). Depending on the quality of the histopathological images, a CNN architecture offers great flexibility and enables a variety of choices. For example, methods have been proposed to work directly with the available resolution if this is low (Postavaru et al., 2017) or on patches from high resolution images with the multiple decisions being further integrated by different techniques (Komura and Ishikawa, 2018). From hand-crafted architectures (Mishra et al., 2018) to pre-trained networks (Araújo et al., 2017) or residual models (Huang and Chung, 2018), CNNs have gained importance in the last few years in their use for tissue segmentation. One area of active application is in diagnosing and understanding breast cancer (Doyle et al., 2008; Abdel-Zaher and Eldeib, 2016; Ehteshami Bejnordi et al., 2017, 2018; Guo et al., 2019; Harvey et al., 2019; Xie et al., 2019). While progress has been made in mimicking traditional processes involving epithelial transformations (Hu et al., 2005; Finak et al., 2008; Beck et al., 2011; Conklin and Keely, 2012; Mao et al., 2013), another avenue to improved diagnoses is the use of the tumor microenvironment (Finak et al., 2008; Hanahan and Coussens, 2012; Mao et al., 2013; Giussani et al., 2018; Jia et al., 2018), both from conventional microenvironment measures and in using emerging imaging techniques (Kumar et al., 2013). In this study, we analyzed breast tissue microarrays (TMA) and surgical specimens to identify different cell signatures in the tumor and its microenvironment using both unsupervised and supervised strategies. First, a deep learning model was built to separate the epithelial, stromal, and other cellular components of the tissue. This allowed for precise investigation of different cellular distributions and their features in each of these components. Next, we evaluated the epithelial and stromal regions as indicators of cancer. Together, we sought to determine common features that expand the palette of digital markers to characterize breast cancer.

## Materials and Methods

### Data Collection

A tissue microarray (TMA) consisting of 100 patient samples, each 1 mm in diameter was obtained from US Biomax Inc. It spans a wide range of disease state i.e. hyperplasia (20 cases), atypical hyperplasia (20), invasive (20 ductal and 20 lobular) and normal (20). This allows for a generalizable model development as it encapsulates a broad range of tumor and cell heterogeneity. These are formalin fixed and paraffin embedded and 5 microns thick samples that are typically used in clinics. Multiple consecutive sections of this TMA were used to stain for different molecular markers. In this study, we have utilized the Hematoxylin and Eosin (H&E) stained images. To take into account sample preparation and staining variability, some tissue sections were also obtained from the breast tissue registry at Washington School of Medicine at St. Louis. All the stained slides were scanned using the Hamamatsu whole slide scanner.

### Data Preparation for Building the CNN Model

Images of whole H&E (hematoxylin and eosin) slides, each corresponding to a different patient, were acquired. The H&E stains are used to identify the morphological changes associated with cancer development and its subsequent progression (Fischer et al., 2008). The data set that is used for training the CNN architecture does not depend on slides to be manually annotated entirely, which would necessitate intensive effort from human experts. However, a pathologist does annotate some sections from each image that correspond to one of the following classes: epithelium (normal, malignant, DCIS), dense stroma, reactive stroma, and the rest in a class that is further referred as *others*. Each contour is chosen to be specific to its class and its size varies from very small regions having an area of <1 mm × 1 mm up to large ones with areas of about 1.5 mm × 1.5 mm. There are 222 such contours annotated and each one of them is used to create image tiles that will further comprise the training, validation and test sets of the CNN model. Squared tiles varying in size from 48 × 48 pixels up to 256 × 256 pixels are cropped from each annotated section. The sizes of the tiles are randomly generated within the mentioned bounds. Random sizes for the cropped images are considered with the aim of feeding the CNN model with sections that contain multiple views of the tissue, from capturing minor details in the smallest squared image to having an overview in the largest squared tile case. The positions of the cropped images are randomly generated such that they lie entirely inside the contour. All the tiles are resized to the minimal size of 48 × 48 pixels when they finally enter the CNN model. Next, a filter is applied to remove tiles with minimal tissue. If the background comprises more than 30% of the tile area, it is not passed to the model. The amount of tiles that are cropped from each annotated contour is proportional to the area of the annotation: the larger the initial area, the more patches are selected, but no more than 100 tiles per annotation. The actual amount of tiles is obtained by dividing the area of the annotated region to the area of a tile with a side of 152 (mean between the minimum 48 and the maximum 256); the double of this division result represents the number of tiles selected for the annotated area. This ensures that a large part of the contour is cropped with overlapping tiles. Also, distinct patients are used for training, validation and test sets for robust classification and unbiased estimates. There are 6 patients for training and 5 others for validation, the amount of tiles generated for the training set is 3914 for the epithelial class, 2578 for stroma and 2122 for others. For the validation set, 1758 patches for epithelium, 1114 for stroma and 414 for others are used. The test set is represented by an independent TMA consisting of 100 patients.

### Tissue Segmentation Pipeline

**Figure 1:**
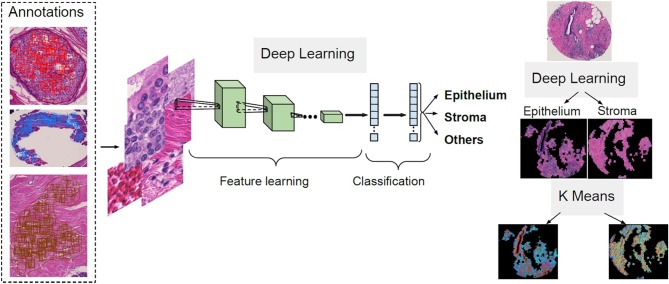
Overview of the proposed deep learning and clustering framework.

The first step of the process for the H&E slides segmentation is the separation between components such as epithelium, stroma and other cellular components. A CNN architecture is employed to achieve this delineation. Next, unsupervised segmentation is applied to both the epithelial and stromal compartments for further investigation in relating to disease. A K-means clustering operation is applied for the pixel values of each component, with the aim of distinguishing between regions in the image that belong to different disease states and the type of cellular moieties present around them. A broad overview of this pipeline is illustrated in Figure 1.

### CNN Model

The goal of the CNN model is to classify large tissue slides into three classes (epithelium, stroma, and others). Thus, each tile will be associated to one of the learned classes and a global matrix of classes is obtained for the entire slide, where each position in the matrix corresponds to a tile from the image. To achieve this, we are faced with the following competing constraints: while the size of the tile should be large enough to contain meaningful information it should also be as small as possible to make the overall tissue image mask look less rasterized and able to delineate borders between classes. To balance these constraints, we resized the tiles to 48 × 48, a size that is considered the minimum acceptable threshold in Keras applications (Chollet, 2017), which are pre-defined deep learning models with pre-trained weights that are known to be useful for prediction as well as feature extraction. Three CNN models, i.e., ResNet50 (He et al., 2015), VGG19 (Simonyan and Zisserman, 2014) and InceptionResNetV2, were implemented to classify the data using transfer learning and default parameters. The results of the three were competitive with image tiles of 197 × 197 pixels as input, with ResNet50 reaching a classification accuracy of 98.74%, VGG19 97.18% and InceptionResNetV2 94.93%. However, VGG19 was kept for further parameter tuning since it conveniently allowed tiles as low as 48 × 48 pixels as input, with the classification accuracy decreasing to 96.14%. Nevertheless, it was considered as a good tradeoff (~1%), since smaller tiles lead to a more refined classification in the larger images.

**Figure 2:**
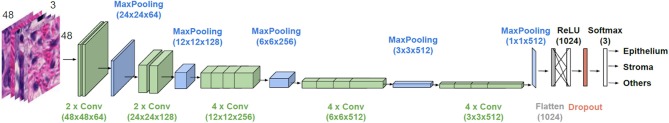
Architecture of the employed CNN model. VGG19 is used for transfer learning. Parameter values are given in parenthesis.

The general architecture of the model used is illustrated in Figure 2. VGG19 has a sequence of several layers that perform convolution (with the given kernel size and number of filters) and max-pooling for down sampling (with the specified filter size and stride). They are followed by a layer that flattens its output volume into a vector to be used as input for the subsequent fully connected layers. A dense layer with a Rectified Linear Unit (ReLU) activation function with 1,024 units continues the suite, followed by a dropout layer with the aim to improve the generalization ability, and consequently decrease overfitting. Finally, a dense layer with a softmax activation function gives the n-dimensional output of the network, given in the form of probabilities of belonging to each class, where n is the number of classes (three in our case). A batch size of 32 images is considered.

The weights of the initial layers are preserved (frozen) from a pre-trained network as they refer to general feature information obtained from learning over a large database. We searched for a number of trainable end layers that is appropriate for learning H&E images and the optimal number was found to be 12. The optimizer to be used for weight adjustment, when minimizing the difference between the predicted output and the ground truth, was another parameter for which we searched for an appropriate solution. The methods of stochastic gradient descent, RMSprop and Adam with various learning rates and decays were explored. The best solutions were found for Adam with a learning rate of 1e-07 and without a decay. The dropout rate, giving the percent of units that are blocked, was also tuned and a value of 0.2 was finally used. The decisions took into account the F1 score (reaching in the best configuration 94.42%) on the validation set and also targeted to minimize overfitting.

Based on the information about general tissue architecture, there are rare cases when scarce tiles belonging to one class will have a neighborhood of other classes. For instance, it is uncommon that a few isolated pixels of epithelium will be surrounded by stroma as epithelial cells are present in ducts or lobules. Therefore, a substantial number of pixels from one class would be present next to an appreciable number of pixels from another, especially in the case of epithelium and stroma. So, we apply a majority filter to our classified images to remove classification noise.

### Clustering

As suggested previously (Beck et al., 2011), it is important to first precisely separate the tissue into the epithelial and stromal compartments for detailed analysis of each of these components for diagnostic or prognostic information. Therefore, we utilize epithelial and stromal regions identified by the deep learning model discussed above. In order to increase the sensitivity of the model, a K-means approach with two clusters is applied to the result obtained to further filter out small regions that were misclassified by the CNN model to only analyze patterns within one cell type at a time. Each detected component is subsequently subject to another K-means clustering algorithm using various number of clusters ranging between 2 and 5. We were interested to measure if there are significant differences between various cluster distributions of each class and how that correlates to distinct disease stages over multiple patients. To achieve this, inertia of both the epithelial and stromal clusters is used. It measures the dispersion of points within a cluster by computing the sum of squared distances for each point to its assigned cluster centroid. A detailed description and code for the CNN model and clustering can be found in the Supplementary Information.

## Results

**Figure 3:**
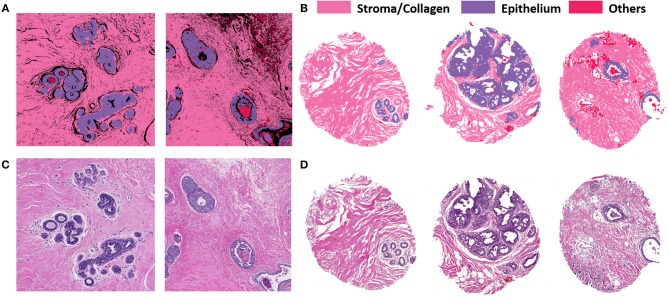
VGG classification on both surgical (training and validation) and tissue microarray (test) samples shows good correspondence with H&E images. **(A)** Classified images of cropped images from two different surgical samples. **(B)** Classified images from three different patients with distinct disease states from the TMA. **(C)** H&E stained images from corresponding surgical samples. **(D)** H&E stained images from the TMA for ground truth comparisons.

Figure 3 illustrates the performance of the developed deep learning strategy for identifying epithelial, stromal and other cellular components (typically consisting of necrosis, red blood cells, secretions and mucin). VGG19 was the method of choice because it allowed tiles of the smaller considered size, while preserving a high classification accuracy. Panel A shows the classification performance on cropped regions from large surgical samples. Surgical resections represent a challenge in terms of size of the sample and data handling, but do provide unambiguous ground truth for disease that may not be present in limited sections from needle biopsies. High accuracy of tissue segmentation is evident by comparison with ground truth H&E stained images (panel C). We also tested our methods on TMAs, which provide the opportunity for a large number of highly diverse samples but recognize that the potential to examine many components of the tumor and microenvironment in each sample may be limited. Figures 3B,D illustrate the model performance on three samples from an independent TMA consisting of 100 patients belonging to different disease states. This array was obtained from a different source to check for model robustness for samples processed and prepared in different institutions or settings. The model performs with similar accuracies on this external validation set. Each sample shown in Panel B (classified images) and Panel D (H&E stained images) belongs to a different patient. The results indicate a good agreement of the histologic units detected by the algorithm (top row) with the ground truth (bottom row). In each case, the prediction was generally good but there were also some small discrepancies as can be seen from the terminal ductal unit in the TMA sample on the left in 3(B) and (D).

**Figure 4:**
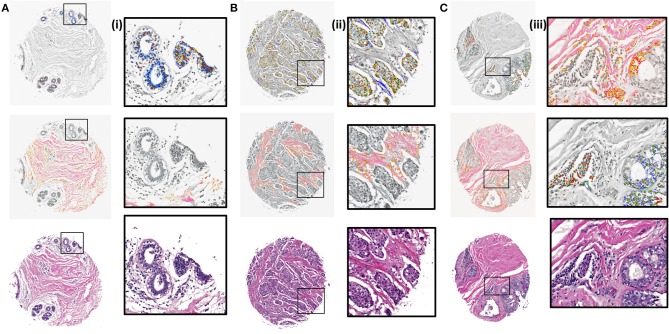
K-means clustering results overlaid on H&E stained images (gray scale) for test samples using 5 clusters. **(A)** Benign case with epithelial clustering (top), stromal clustering (middle) and H&E stained image (bottom). (i) Corresponding zoomed in views from images in **(A)**. **(B)** Ductal carcinoma case with epithelial and stromal clustering along with the stained image. (ii). Zoomed in views from images in **(B)**. **(C)** Lobular carcinoma case with clustering and ground truth comparison. (iii). Zoomed regions from images in **(C)**.

Next, K-means images for the components identified by the CNN model were examined. The clustering algorithm is run for 300 iterations, 10 times with random seeds each time and the best solution is kept. It is applied to the RGB values of the masked images based on the CNN results. The clustered images with 5 clusters were overlaid with grayscale H&E stained images to illustrate the class distributions along with a reference to the tissue architecture. Different colors in the overlaid images represent different clusters that are indicative of either different cellular types or subtypes within a cell type. It is evident from zoomed views in Figure 4 (i), (ii), and (iii) that different signatures within the epithelial (top) and stromal (middle) regions are identified. It can also be noted [Figure 4 (ii), middle section] that cellular structures like lymphocytes, fibroblasts and plasma cells that are typically present in the stroma get highlighted in the stromal clustering as a different class i.e., a different color. This is important, as sometimes these components can be confused with epithelial cells and these cells constitute an important part of the tumor microenvironment. Our method eliminates the confusion between epithelial and stromal cells by this two-tiered approach and provides a means to discover less abundant cells in the microenvironment. The challenge remains to harness these signatures of different components obtained by unsupervised discovery for diagnosis. While this work focuses on providing a viable digital pathway, careful curation and labeling of microenvironment components in many surgical samples will be needed to further refine this approach.

**Figure 5:**
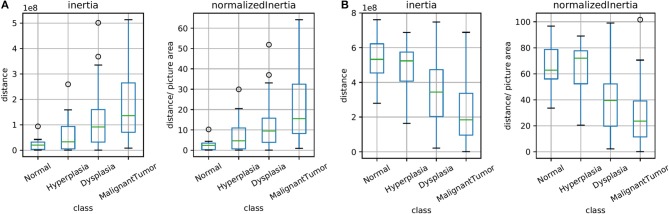
Box plots for the calculated inertia (dispersion of points within a cluster) over all TMA images and for inertia divided by the image area (normalized), both for epithelium in **(A)** and stroma in **(B)**. This illustrates the extent of differentiation in the epithelium and stromal compartments for different levels of disease states.

The unsupervised clusters are indicative of potential changes in the microenvironment. Here, we sought to explore whether the clusters have any bearing on diagnoses of disease. Figure 5 illustrates the spread of clusters (inertia) in the epithelial and stromal compartments for different disease states. Inertia is the within cluster sum of squares indicative of intra cluster coherence. For the epithelial distribution in Figure 5A, the average inertia for the malignant class is the highest and the normal class is the lowest. It is interesting that the intraductal proliferations (Ellis, 2010) (hyperplasia and dysplasia) also follow the same trend with the high risk group (dysplasia) having a higher mean inertia and the low risk group (hyperplasia) having a lower inertia. This can help in increasing confidence of the diagnostic decision especially since the histology criteria can be subjective and in some cases not clearly defined. Our hypothesis is that disruption of normal physiologic structure of the tissue results in a difference in inertia, either by decreased order in consistent structures (e.g., changes in epithelial morphology) or imposition of homogeneity on well-differentiated functional units of tissue (e.g., tissue transitioning from clear functional units in a well-defined differentiated pattern to becoming poorly differentiated in space). This hypothesis can be used as a basis for designing digital features that indicate disease. Though a single feature is not unambiguous for any given sample, the trends are useful in adding value to automated methods, provide a comforting validation of designing digital markers using hypotheses of organization and provide potential for further refinement.

**Figure 6:**
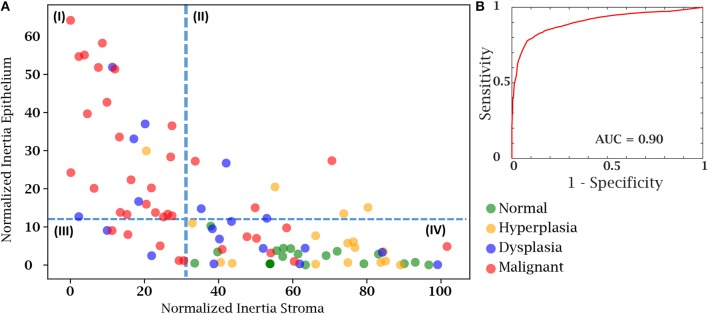
Cancer detection using both the epithelial and stromal spatial distributions. **(A)** Scatter plot separating patients from different disease states based on normalized inertia. **(B)** Receiver Operating Characteristic (ROC) curve of using inertia as a cancer detection tool. The decision boundary shown in the figure is an illustration of one of the points on the ROC curve. All patients belonging to the fourth quadrant are labeled as normal.

Finally, we sought to use both the epithelial and stromal inertia together to examine whether they provided diagnostic value. From the total of 100 cases in our test case, 8 patient cases either had insufficient epithelial or stromal yield. These were discarded as the epithelium and stromal inertia couldn't be compared. Therefore, we confirmed our hypothesis using an independent set of 92 patient cases belonging to different disease states. We compared the normalized inertia of the epithelial and stromal compartments. By considering a cut-off line for each of the epithelial and stromal figures of merit, we obtain two intersecting lines that divide the entire distribution of inertia into four quadrants. It is evident from Figure 6A that the normal and hyperplasia cases are mostly in quadrant IV and malignant cases are distributed in all the other quadrants with a majority lying in the first quadrant. The dotted lines can also be used as a threshold to separate cancer from the normal cases. A variety of 2D thresholds can be used and the receiver operating characteristic curve in Figure 6B shows the sensitivity and specificity profile of using inertia as a measure of detecting malignancy. For estimating the ROC curve, the malignant cases are labeled as the cancer class and all other categories i.e., hyperplasia, dysplasia, and normal are combined together into the non-cancer class. The area under the curve is a measure of the model performance and the closer it is to 1, the better the model. The high AUC values suggest that a combinatory epithelial and stromal approach to extract spatial features (inertia in this case) is a good indicator of disease and its progression. Though, again, not a perfect discriminator, the result provides a useful means to utilize the microenvironment and ensure that the results are consistent with underlying diagnoses. Most importantly, the work paves the way for further assessment of complex cellular features in both epithelial cells as well as different stromal cells. Distribution of these cells and other spatial measures of the tumor can provide a further boost in accuracy to the method developed here. An advantage of the proposed method is that it is simple to understand and easy to interpret. Unlike a typical deep learning approach in which images are the input and a decision is the output, this tiered approach provides an insight to understand and interpret some of the vast information encoded in tissue on disease states.

## Conclusions

This paper presents a deep learning scheme for digital tissue segmentation using stained images which can further be used to extract sophisticated spatial features for cancer diagnosis in an automated, fast and accurate manner. Our approach relied on a tiered understanding of tissue structure as deep learning methods were applied. First, we segmented tissue into epithelial and stromal compartments. Second, we examined the heterogeneity in the stroma using clustering, which resulted in separation of tissue microenvironment components like immune cells and cancer associated fibroblasts. Finally, we sought to use a simple and interpretable characterization of the tissue in terms of inertia to recognize disease. The work establishes the concept of multidimensional inertia to be used as an indicator of disease and its outcome, without needing any additional resources or disruption to the current workflow. The analytics tool can be integrated with whole slide scanners for interactive and real time sample analysis to aid human experts. This study focused on understanding the power of simultaneously using the epithelial and stromal signatures to separate different diseased states. This needs to be further tested in supervised classification schemes along with other tissue predictors. With further refinement of this approach, more spatial signatures and detailed understanding of the tumor and the microenvironment can lead to increased insight into digital characterization of breast cancer using conventionally stained images.

## Data Availability Statement

The datasets generated for this study are available on request to the corresponding author.

## Ethics Statement

This study used de-identified human subject specimens. All protocols were performed according to the approved project by the University of Illinois at Urbana-Champaign Institutional Review Board i.e., IRB 06684.

## Author Contributions

SM and RB designed the research. SM and AK-B collected and annotated the data. CS and SM performed the computational analysis. SM, CS, and RB wrote the manuscript.

### Conflict of Interest

The authors declare that the research was conducted in the absence of any commercial or financial relationships that could be construed as a potential conflict of interest.
