# Supplementary material for: Digital Assessment of Stained Breast Tissue Images for Comprehensive Tumor and Microenvironment Analysis
Source: Front Bioeng Biotechnol. 2019 Oct 1;7:246. doi: 10.3389/fbioe.2019.00246 (PMC6797859; doi:10.3389/fbioe.2019.00246)
Supplement: Supplementary file 1 [file Data_Sheet_1.PDF]

Source Code - Digital Assessment of Stained Breast Tissue Images for Comprehensive Tumor and Microenvironmental Analysis

April 16, 2019

1 Overview

1.1 CNN Model

In order to build the CNN model, we need to have crops for Epithelium, Stroma and for Others. Crops will be saved in folders having the following structure:

- train
  - Epithelium
  - Stroma
  - Others
- validation
  - Epithelium
  - Stroma
  - Others
- test
  - Epithelium
  - Stroma
  - Others

The variables *trainPath*, *validPath* and *testPath* will be initialized with the paths to the corresponding folders above. A model will be built and saved for future references when building colored masks of the original images.

1.2 Colored Masks

The original large H&E images are saved in the folder names *DatasetTileClassification*. For each 48x48 pixels tile in these we will create colored masks that correspond to *Epithelium*, *Stroma* and *Others*. The colored masks will have the following colors: - Epithelium = dark green - Stroma = red - Others = blue - Background = black

A tile is considered to be in background if up to 90% of it is very light (pixel values are above 200). See *goodTile* method for details.

Having the colored masks computed, these can be transformed to gray scale and each component can be obtained by specific thresholds as follows: * 76 = Stroma * 58 = Epithelium * 29 = Others

Each original H&E image will have a corresponding colored mask in folder *ColoredMasks*. When the run starts, first check the ColoredMasks folder to see if the current file has the colored mask already computed. This way, no time is lost with applying the VGG19 model to the initial image. All the initial images will have a colored mask saved in ColoredMasks folder with the name of the file followed by ColoredMask.jpg

1.3 K Means

We will verify first if there are already results in the SavedResults folder for noOfClusters and Stroma or Epithelium, depending on the threshold. K Means is applied either for Stroma or for Epithelium by taking the appropriate mask from the initial H&E image. For each one, there are several results saved in a *.csv* file: file name, inertia, w, h, w * h, number of pixels for stroma, number of pixels for epithelium, number of pixels for others, number of pixels for background, number of pixels overall, the same amounts in percents, the values x1, x2 and x3 for every cluster, class of the image, running time K Means, running time for making the colored masks, running time overall.

2 Building the CNN model

```
In [ ]: from keras.preprocessing.image import ImageDataGenerator
        from keras.models import Sequential
        from keras.layers import Dropout, Flatten, Dense
        from keras import optimizers
        from keras.applications import VGG19
        from keras.callbacks import ModelCheckpoint, TensorBoard
        from keras import backend

        import os
        import glob #to count jpg files

        import numpy as np

        import matplotlib.pyplot as plt
        %matplotlib inline

        import time #to measure running time

        def runCNN(noOfRuns = 1, epochs = 100, batch_size = 32, image_size = 197):
            for i in range (0, noOfRuns):
                start_time = time.time()
                print("Starting run ", i)
                backend.clear_session()

                res_conv = VGG19(weights='imagenet', include_top=False,
```

```python
                 input_shape=(image_size, image_size, 3))

# Freeze the layers except the last 4 layers
for layer in res_conv.layers[:-4]:
    layer.trainable = False

# Create the model
model1 = Sequential()

# Add the res convolutional base model
model1.add(res_conv)

# Add new layers
model1.add(Flatten())
model1.add(Dense(1024, activation='relu'))
model1.add(Dropout(0.2))
model1.add(Dense(3, activation='softmax'))

# Show a summary of the model. Check the number of trainable parameters
model1.summary()

# this is the augmentation configuration we will use for training
train_datagen = ImageDataGenerator(rescale=1./255,
                      rotation_range=20,
                      width_shift_range=0.2,
                      height_shift_range=0.2,
                      horizontal_flip=True,
                      fill_mode='nearest')

# only rescaling for validation and test
valid_datagen = ImageDataGenerator(rescale=1./255)
test_datagen = ImageDataGenerator(rescale=1./255)
seed = 5

# this is a generator that will read pictures found in
# subfolDers of 'data/train', and indefinitely generate
# batches of augmented image data
train_generator = train_datagen.flow_from_directory(
        trainPath,  # this is the target directory
        target_size=(image_size, image_size),
        batch_size=batch_size,
        class_mode='categorical', seed=seed, shuffle = True)

# this is a similar generator, for validation data
validation_generator = valid_datagen.flow_from_directory(
        validPath,
        target_size=(image_size, image_size),
        batch_size=batch_size,
```

```
                class_mode='categorical', shuffle = False)

        test_generator = test_datagen.flow_from_directory(
                testPath,
                target_size=(image_size, image_size),
                batch_size=jpgCounterTest,
                class_mode='categorical', shuffle = False)

        filepath="D:\\SavedResults\\3ClassesVGG19TuneAugmentation" + str(i) +".hdf5"

        checkpoint = ModelCheckpoint(filepath, monitor='val_acc',
                                     verbose=1, save_best_only=True, mode='max')

        #use tensorboard to check the runs' outputs live
        tbCallBack = TensorBoard(log_dir='Graph', histogram_freq=0,
          write_graph=True, write_images=True)
        tbCallBack.set_model(model1)

        callbacks_list = [checkpoint, tbCallBack]
        #callbacks_list = [tbCallBack]
        model1.compile(loss='categorical_crossentropy',
                      optimizer=optimizers.Adam(lr=1e-07),
                      metrics=['acc'])

        history2 = model1.fit_generator(
                train_generator,
                #steps_per_epoch=100,#2000 // batch_size,
                epochs=epochs,
                validation_data=validation_generator,
                callbacks=callbacks_list,
                validation_steps=jpgCounterValid // batch_size)
        elapsedTrainingTime = time.time() - start_time
        print('Run {} is done in {} seconds'.format(i + 1, elapsedTrainingTime))

    return

trainPath = 'D:\\Data3Classes\\train'
validPath = 'D:\\Data3Classes\\valid'
testPath = 'D:\\Data3Classes\\test'


jpgCounterValid = len(glob.glob(validPath + "/**/*.tif"))
                + len(glob.glob(validPath + "/**/*.jpg"))
jpgCounterTest = len(glob.glob(testPath + "/**/*.tif"))
                + len(glob.glob(testPath + "/**/*.jpg"))

runCNN(noOfRuns = 1, epochs = 5, batch_size = 32, image_size = 48)
```

3 Load the built model and use it to predict the outcome of one image

In [ ]:
```
from keras.applications import VGG19
from keras.preprocessing import image
from keras.models import Sequential
from keras.layers import Activation, Dropout, Flatten, Dense

def constructVGGModel():
    dx=dy=48
    modelPath = "D:\\SavedResults\\3ClassesVGG19TuneAugmentation0.hdf5"

    res_conv = VGG19(weights='imagenet', include_top=False, input_shape=(dx, dy, 3))


    # Freeze the layers except the last 12 layers
    for layer in res_conv.layers[:-12]:
        layer.trainable = False

    # Create the model
    model1 = Sequential()

    # Add the res convolutional base model
    model1.add(res_conv)

    # Add new layers
    model1.add(Flatten())
    model1.add(Dense(1024, activation='relu'))
    model1.add(Dropout(0.2))
    model1.add(Dense(3, activation='softmax'))

    model1.load_weights(modelPath)

    return model1

def predictImage(test_image):
    test_image = keras.preprocessing.image.img_to_array(test_image)
    test_image = test_image/255
    test_image = np.expand_dims(test_image, axis = 0)
    result = model1.predict(test_image)
    return result.argmax(1)

model1 = constructVGGModel()
```

4 Methods for getting the class of an image based on the patient ID

In [3]:
```
######################
# These classes depend a lot on the data set and the names of the files.
```

```
# They will need to be adapted to thedata set.
######################

mainPath = "D:\\"
# INPATH contains the initial H & E files for which the K Means in applied
INPATH = mainPath + "DatasetTileClassification"

#getClass returns a string with the class of the current
#patient based on the patient ID number
#the classes for them are known in advance
def getClass(fileName):
    classFound = False
    i = 0
    currentClass = 'notFound'
    while i < len(patientIDs) and classFound == False:
        if patientIDs[i] in fileName:
            currentClass = patientClasses[i]
            classFound = True
        i = i + 1

    return currentClass

def getClass(fileName):
    classFound = False
    i = 0
    currentClass = 'notFound'
    while i < len(patientClasses) and classFound == False:
        if patientIDs[i] in fileName:
            currentClass = patientClasses[i]
            classFound = True
        i += 1

    return currentClass


def getPatientID(fileName):
    classFound = False
    i = 0
    currentPatientID = 'notFound'
    while i < len(patientIDs) and classFound == False:
        if patientIDs[i] in fileName:
            currentPatientID = patientIDs[i]
            classFound = True
        i += 1

    return currentPatientID

'''
```

```python
We need to name the fileNames with ColXRowY, where X and Y starts from 10.
The patient tissues are here: https://www.biomax.us/tissue-arrays/Breast/BR1003
'''
def defineClassesAndIDs():
    patientIDs = []
    for i in range(10):
        for j in range(10):
            patientIDs.append('Col' + str(j) + 'Row' + str(i))
    patientIDs.append('Col10Row9')
    patientClasses = []
    for i in range(10):
        for j in range(2):
            patientClasses.append('Hyperplasia')
    for i in range(10):
        for j in range(2,4):
            patientClasses.append('Dysplasia')
    for i in range(10):
        for j in range(4,8):
            patientClasses.append('MalignantTumor')
    for i in range(10):
        for j in range(8,10):
            patientClasses.append('Normal')
    patientClasses.append('Normal')

    patientIDs += ['42289', '42312', '43027', '43028',
                   '43059', '43069', '43075', '43290',
                   '43292', '43334', '43474', '43495',
                   '43505', '44023', '43434', '43025',
                   '43035', '43319', '43320', '43036',
                   '43026', '43318', '44022', '44024',
                   '43024', '43034', '44025']
    patientClasses += ['DCIS', 'DCIS', 'DCIS', 'DCIS',
                       'DCIS', 'DCIS', 'DCIS', 'IBC',
                       'Normal', 'IBC', 'IBC', 'IBC',
                       'IBC', 'IBC', 'DCIS', 'DCIS',
                       'DCIS', 'MalignantTumor', 'Normal',
                       'Normal', 'Normal', 'DCIS', 'DCIS',
                       'MalignantTumor', 'DCIS', 'DCIS', 'Normal']

    return patientIDs, patientClasses

def findActualPatient(fileName):
    equivalenceClasses = [[43319, 43318, 43320],
                           [43035, 43034, 43036],
                           [43025, 43024, 43026],
                     [44023, 44022, 44024, 44025]]
    actualID = -1
    foundID = False
```

```
    i = 0
    while i < len(equivalenceClasses) and foundID == False:
        j = 0
        while j < len(equivalenceClasses[i]) and foundID == False:
            if str(equivalenceClasses[i][j]) in fileName:
                foundID = True
                actualID = equivalenceClasses[i][0]
            j += 1
        i += 1
    return actualID

def getOriginalFileName(file):
    originalFiles = os.listdir(INPATH)
    numOfOriginalImages = len(originalFiles)
    classFound = False
    i = 0
    originalFileName = 'notFound'
    while i < numOfOriginalImages and classFound == False:
        if originalFiles[i] in file:
            originalFileName = originalFiles[i]
            classFound = True
        i += 1
    return originalFileName
```

5 Methods for labelling the crops in a large image

```
In [5]: import time
        import os
        from PIL import Image, ImageFont, ImageDraw, ImageEnhance
        import matplotlib.pyplot as plt
        import matplotlib.image as mpimg
        import re #to get a substring between 2 strings

        import collections# to count frequencies in arrays

        import cv2
        from sklearn.cluster import KMeans
        import numpy as np

        Image.MAX_IMAGE_PIXELS = 1000000000 #in order to allow very large images as input

        '''
        The inputs are:
        * DataColoredTileClassification = the original images
        * ColoredMasks = the masks with 3 colors for the original images.
            The colors correspond to stroma, epithelium and others.
        '''
```

```
'''
Gets the most common element from a list.
Used for cleaning the colored mask image for epithelium/stroma/others
'''
def mostCommon(lst):
    return max(set(lst), key=lst.count)

'''
Receives a matrix of predictions and returns a
matrix in which the singleton classes
are replaced by the common classes around them.
'''
def cleanMatrixClasses(matrix):
    m = matrix
    X, Y = matrix.shape

    classesAround = lambda x, y : [matrix[x2][y2] for x2 in range(x-1, x+2)
                                   for y2 in range(y-1, y+2)
                                   if (-1 < x < X and
                                       -1 < y < Y and
                                       (x != x2 or y != y2) and
                                       (0 <= x2 < X) and
                                       (0 <= y2 < Y))]

    for j in range(Y):
        for i in range(X):
            if len(classesAround(i, j)) == 3: #if it is in the corner
                #if there no similar tile
                if classesAround(i, j).count(matrix[i][j]) == 0:
                    m[i][j] = mostCommon(classesAround(i, j))
            else: #there are 5 or 8 neighbors
                #if there one single tile or 0, change it to the majority
                if classesAround(i, j).count(matrix[i][j]) < 3:
                    m[i][j] = mostCommon(classesAround(i, j))
    return m

def drawColoredRectangles(draw, x, y, w, h, predictedOutput):
    if predictedOutput == -1:
        draw.rectangle([(x,y),x + dx, y + dy], fill = 'black' )
    elif predictedOutput == 0:
        draw.rectangle([(x,y),x + dx, y + dy], fill = 'darkgreen' )
    elif predictedOutput == 1:
        draw.rectangle([(x,y),x + dx, y + dy], fill = 'blue' )
    elif predictedOutput == 2:
        draw.rectangle([(x,y),x + dx, y + dy], fill = 'red' )
    return draw
```

```
#a tile is good if at least percentPixel% are below 200,
#that is they are not only white
#uses PIL as input
def goodTile(im, percentPixels = 90):
    good = True
    pix = np.array(im, dtype=np.float)
    mask = (pix>200).all(axis=-1)
    pix[mask] = 1

    w, h = mask.shape

    if (np.sum(mask) / (w * h)) > (percentPixels / 100):
        good = False
    return good

'''
Get the original image, make a mask that identifies light pixels (>200),
add this mask to the colored mask image and return the obtained image.
'''
def combineOriginalAndMaskToEliminateWhite(imOrig, imMask):
    pix = np.array(imOrig, dtype=np.float)
    pixMask = np.array(imMask, dtype=np.float)
    mask = (pix>200).all(axis=-1)
    pixMask[mask] = 1
    return Image.fromarray(np.uint8(pixMask))

#gets an image and returns the pixels that are above 200 transformed in black
def getImageWithoutWhite(im):
    pix = np.array(im, dtype=np.float)

    mask = (pix>200).all(axis=-1)
    pix[mask] = 1

    return Image.fromarray(np.uint8(pix))


'''
Receives a fileName and makes a matrix of classes for it.
'''
def makeClassMatrixForOneFile(fileName):
    startImagePred = time.time()
    im = Image.open(fileName)

    w, h = im.size

    timesInWidth = int(w / dx) + (w % dx > 0)
    timesInHight = int(h / dy) + (h % dy > 0)
```

```python
noOfTilesTotal = timesInWidth * timesInHight
print('%.2f times in width and %.2f in height, ',
      'that is %.2f applications for the main ',
      'part.' % (timesInWidth, timesInHight, noOfTilesTotal))
print('This will take approx %.2f seconds, ',
      'that is %.2f minutes.' % ((noOfTilesTotal * 0.02),
                                 ((noOfTilesTotal * 0.02) / 60)))

matrixOfClasses = np.zeros((timesInWidth, timesInHight))
x = 0
y = 0

x1 = 0
y1 = 0

while x < w - dx:
    while y < h - dy:
        croppedImage = im.crop((x, y, x + dx, y + dy))
        if goodTile(croppedImage):
            predictedOutput = predictImage(croppedImage)
        else:
            predictedOutput = -1 #will be colored as black
        matrixOfClasses[x1][y1] = predictedOutput
        y = y + dy
        y1 += 1
    x = x + dx
    x1 += 1
    maxSizeY = y1
    y = 0
    y1 = 0

#take care of the margins
x = 0
y = h - dy

x1 = 0
y1 = timesInHight - 1

while x < w - dx:
    croppedImage = im.crop((x, y, x + dx, y + dy))
    if goodTile(croppedImage):
        predictedOutput = predictImage(croppedImage)
    else:
        predictedOutput = -1 #will be colored as black
    matrixOfClasses[x1][y1] = predictedOutput
    x = x + dx
    x1 += 1
```

```
    y = 0
    x = w - dx
    x1 = timesInWidth - 1
    y1 = 0
    while y < h - dy:
        if goodTile(croppedImage):
            predictedOutput = predictImage(croppedImage)
        else:
            predictedOutput = -1 #will be colored as black

        predictedOutput = predictImage(croppedImage)
        matrixOfClasses[x1][y1] = predictedOutput
        y = y + dy
        y1 += 1

    #take care of the final corner-down
    x = w - dx
    y = h - dy
    x1 = timesInWidth - 1
    y1 = timesInHight - 1
    if goodTile(croppedImage):
        predictedOutput = predictImage(croppedImage)
    else:
        predictedOutput = -1 #will be colored as black

    predictedOutput = predictImage(croppedImage)
    matrixOfClasses[x1][y1] = predictedOutput

    endImagePred = time.time()

    matrixOfClasses = matrixOfClasses.astype(int)

    return matrixOfClasses, endImagePred - startImagePred;

'''
Next method receives the fileName and the matrix
to make the colored mask.
'''
def makeColoredMaskFromMatrix(fileName, m):
    startImagePred = time.time()
    im = Image.open(fileName)
    x = 0
    y = 0

    w, h = im.size
    timesInWidth = int(w / dx) + (w % dx > 0)
    timesInHight = int(h / dy) + (h % dy > 0)
```

```python
noOfTilesTotal = timesInWidth * timesInHight
maskImage = Image.new("RGB", (w, h), (0, 0, 0))
draw = ImageDraw.Draw(maskImage)

x = 0
y = 0

x1 = 0
y1 = 0

while x < w - dx:
    while y < h - dy:
        drawColoredRectangles(draw, x, y, x + dx, y + dy, m[x1][y1])
        y = y + dy
        y1 += 1
    x = x + dx
    x1 += 1
    maxSizeY = y1
    y = 0
    y1 = 0

#take care of the margins
x = 0
y = h - dy

x1 = 0
y1 = timesInHight - 1

while x < w - dx:
    drawColoredRectangles(draw, x, y, x + dx, y + dy, m[x1][y1])
    x = x + dx
    x1 += 1

y = 0
x = w - dx
x1 = timesInWidth - 1
y1 = 0
while y < h - dy:
    drawColoredRectangles(draw, x, y, x + dx, y + dy, m[x1][y1])
    y = y + dy
    y1 += 1

#take care of the final corner-down
x = w - dx
y = h - dy
x1 = timesInWidth - 1
y1 = timesInHight - 1
drawColoredRectangles(draw, x, y, x + dx, y + dy, m[x1][y1])
```

```python
    endImagePred = time.time()

    return maskImage, endImagePred - startImagePred;

'''
Gets the initial H&E files for which the K Means in
applied and the clustered images are saved.
It also checks if the clustered images are
already produced to avoid overwork.
'''
def makeColoredMasks(inputFolder):
    files = os.listdir(inputFolder)
    numOfImages = len(files)
    print('There are ', numOfImages,
          ' to process in the input folder: ', inputFolder)
    for file in files:
        # Search for fileColoredMask in mainPath + 'ColoredMasks'.
        # If there is not such file, make it and save it there
        filesWithMasks = os.listdir(mainPath + 'ColoredMasks')
        classFound = False
        i = 0
        while i < len(filesWithMasks) and classFound == False:
            if file in filesWithMasks[i] and 'Mask' in filesWithMasks[i]:
                classFound = True
            i += 1
        if classFound == True:
            print('File ', file, ' already has a colored mask.')
        else:
            print('Making the colored mask for ',
                  file, ', starting at ', time.ctime())
            start = time.time()

            matrix, runtimeMatrix = makeClassMatrixForOneFile(
                os.path.join(inputFolder, file))

            maskImage, runtimeMaskImage = makeColoredMaskFromMatrix(
                os.path.join(inputFolder, file), cleanMatrixClasses(matrix))


            imOrig = Image.open(os.path.join(inputFolder, file))
            imOrig.save(mainPath + 'ColoredMasks\\' + file + getClass(file)
                        + str(runtimeMatrix + runtimeMaskImage) + 'sec.jpg')

            obtainedMaskImage = combineOriginalAndMaskToEliminateWhite(
                imOrig, maskImage)

            obtainedMaskImage.save(
```

```
                    mainPath + 'ColoredMasks\\' + file + getClass(file)
                    + 'ColoredMask' + str(runtimeMatrix
                    + runtimeMaskImage) + 'sec.tif')

                #plot the initial image
                originalImg = cv2.imread(os.path.join(inputFolder, file))
                originalImg = cv2.cvtColor(originalImg, cv2.COLOR_BGR2RGB)
                imgplot = plt.imshow(originalImg)
                plt.show()

                plt.imshow(obtainedMaskImage)
                plt.show()

                endTime = time.time()
                print('Producing the mask for ', file,
                      ' took ', (endTime - start), ' seconds.')
        return

    def remakePictureWithClusters(maskedImage, clusterImage):
        maskedImage2 = maskedImage.reshape(
            (maskedImage.shape[0] * maskedImage.shape[1], 3))
        mask = np.logical_not(np.all(maskedImage2 == [0, 0, 0], axis = -1))
        maskedImage2[mask] = clusterImage
        maskedImage2 = maskedImage2.reshape(
            (maskedImage.shape[0], maskedImage.shape[1], 3))
        plt.imshow(maskedImage2)
        plt.show()
        return maskedImage2
```

6 Methods for counting pixels of different types

```
In [6]: def countPixelsThresh(imMask, thresh):
            imMask=imMask.convert('L')
            w, h = imMask.size
            fn = lambda x : 1 if x == thresh else 0
            binaryImage = imMask.point(fn, mode='1')
            I = np.asarray(binaryImage)
            return np.sum(I)

        def countPixelsPerType(imMask):
            #count stromaPixels
            pixelsStroma = countPixelsThresh(imMask, 76)
            pixelsEpithelium = countPixelsThresh(imMask, 58)
            pixelsOthers = countPixelsThresh(imMask, 29)
            pixelsBackground = countPixelsThresh(imMask, 1) + countPixelsThresh(imMask, 0)

            return pixelsStroma, pixelsEpithelium, pixelsOthers, pixelsBackground
```

```
def countPixels(imMask):
    pix = np.array(imMask, dtype=np.float)
    differentThanBlack = (pix>1).all(axis=-1)
    w, h = mask.shape

    return np.sum(differentThanBlack), w*h
```

7 Method for plotting the cluster colors and the number of points/cluster

```
In [7]: '''
        Define a barchart that will show how many points
        from each cluster are there and what are their colors.
        This is used only to have an intuitive illustration of the clusters and colors.
        '''
        def getBarChart(
            originalColors, colorNames, sizes, title, noOfClusters, saveFileName):

            index = np.arange(noOfClusters)
            bar_width = 0.35

            xNames = []
            for i in range(noOfClusters):
                xNames.append('C' + str(i + 1))

            hexColors = []
            for i in range(noOfClusters):
                hexColors.append('#%02x%02x%02x' % (colorNames[i][0],
                                     colorNames[i][1], colorNames[i][2]))

            hexColors2 = []
            for i in range(noOfClusters):
                hexColors2.append('#%02x%02x%02x' % (originalColors[i][0],
                                 originalColors[i][1], originalColors[i][2]))


            plt.bar(index, sizes, bar_width, align='center', color = hexColors2)
            plt.bar(index + bar_width, sizes,
                    bar_width, align='center', color = hexColors)
            plt.xticks(index + bar_width/2, xNames)
            plt.ylabel(title)
            plt.tight_layout()

            plt.savefig(saveFileName)

            return
```

8 Main part of the program

```
In [ ]: '''
        In grayscale:
        * 76 = Stroma
        * 58 = Epithelium
        * 29 = Others
        '''

        thresh = 76 #76 the gray value for stroma
        noOfClusters = 5
        classInvestigated = 'Stroma'
        if thresh == 58:
            classInvestigated = 'Epithelium'

        patientIDs, patientClasses = defineClassesAndIDs()

        print('Run started at: ', time.ctime())
        dx = dy = 48
        makeColoredMasks(INPATH) #this transforms the original images into colored masks
        print('All the input images have colored masks.')

        files = os.listdir(mainPath + 'ColoredMasks')
        numOfImages = len(files)
        for file in files:
            originalFileName = getOriginalFileName(file)
            if originalFileName != 'notFound':
                for noOfClusters in range(2,3):
                    resultsAlreadyExist = False
                    #verify if there is result for the current thresh and noOfClusters
                    verifyResultsPath = (mainPath + 'SavedResults\\' +
                                classInvestigated + '\\')

                    verifyResultsPath = verifyResultsPath + 'K = ' + str(noOfClusters)
                    if not os.path.exists(verifyResultsPath):
                        os.makedirs(verifyResultsPath)

                    #create the first header line to add to the clusterResults.csv file
                    firstLine = 'fileName\tinertia\tw\th\tw*h',
                    '\tpointsStroma\tpointsEpithelium',
                    '\tpointsOthers\tpixelsBackground',
                    '\tpointsAll\tpointsStromaPercent',
                    '\tpointsEpitheliumPercent\tpointsOthersPercent',
                    '\tpixelsBackgroundPercent\t'

                    for i in range(0, noOfClusters):
                        firstLine = (firstLine + 'x' + str(i + 1) + '_1\t' + 'x' + str(i + 1)
                                    + '_2\t' + 'x' + str(i + 1) + '_3\t')
```

```
for i in range(0, noOfClusters):
    firstLine = firstLine + 'pointsCluster' + str(i) + '\t'

firstLine = (firstLine + 'class\truntimeKMeans',
        '\truntimeImagePreds\truntimeAllImage')

if os.path.isfile(verifyResultsPath
                  + '\\originalClusterResults'
                  + str(noOfClusters) + '.csv') == False:
    f = open(verifyResultsPath
             + '\\originalClusterResults'
             + str(noOfClusters) + '.csv','w')
    f.write(firstLine)
    f.close()
folderForCurrentImage = (verifyResultsPath
                + '\\P' + getPatientID(file)
                + '_' + getClass(file) + '\\')
#if the folder exists, check if results for the current file exist
if os.path.exists(folderForCurrentImage):
    existingResultFiles = os.listdir(folderForCurrentImage)
    j = 0
    while j < len(existingResultFiles) and resultsAlreadyExist == False:
        if file in existingResultFiles[j]:
            resultsAlreadyExist = True
        j = j + 1
if resultsAlreadyExist or 'Mask' not in file:
    print('There are already results for ', file,
          '. They can be found in ',
          verifyResultsPath, ' or the file is not a mask.')
else:
    print('\nProcessing ', file, ', starting at ', time.ctime())
    start = time.time()
    with Image.open(
        os.path.join(mainPath + 'ColoredMasks', file)) as maskImage:

        maskImage=maskImage.convert('L')

        print('Create the binary image in which the '
              + classInvestigated + ' is identified for ', file)

        fn = lambda x : 1 if x == thresh else 0
        binaryImage = maskImage.point(fn, mode='1')

        originalImg = cv2.imread(INPATH + '\\' + originalFileName)

        open_cv_image = np.array(originalImg, dtype=np.uint8)

        binaryImage2 = np.array(binaryImage, dtype=np.uint8)
```

```
resultImage = cv2.bitwise_and(
    open_cv_image, open_cv_image, mask = binaryImage2)


if not os.path.exists(folderForCurrentImage):
    os.makedirs(folderForCurrentImage)

cv2.imwrite(folderForCurrentImage + '\\'
            + file + 'Mask.jpg', resultImage)#save the mask
#save the original file for easy comparison
cv2.imwrite(folderForCurrentImage
            + '\\Original' +  file, originalImg)

# remove the black from the K Means
img2 = resultImage[np.logical_not(
    np.all(resultImage == [0, 0, 0], axis = -1))]


pixelsStroma, pixelsEpithelium,
pixelsOthers, pixelsBackground = countPixelsPerType(
    Image.fromarray(np.uint8(maskImage)))
pixelsTotal = (pixelsStroma + pixelsEpithelium
                + pixelsOthers + pixelsBackground)

w, h = maskImage.size
pixCount = pixelsStroma
if thresh == 58:
    pixCount = pixelsEpithelium
if pixCount > noOfClusters:

    if pixelsTotal != (w * h):
        print('Not OK! Counting the pixels for stroma ',
              pixelsStroma, ', epithelium', pixelsEpithelium,
              ', others', pixelsOthers, ', background',
              pixelsBackground, ', in total', pixelsTotal,
              ' does not equal w*h', w, '*', h,'=', (w*h))
    else:
        print('Counting the pixels for stroma ', pixelsStroma,
              ', epithelium', pixelsEpithelium, ', others',
              pixelsOthers, ', background', pixelsBackground,
              ', in total', pixelsTotal,
              ' equals w*h', w, '*', h,'=', (w*h))

    print('Apply K-Means for ', file, ' having  resolution of ',
          resultImage.shape, ' and K = ', noOfClusters)
    startK = time.time()
    clt = KMeans(n_clusters = noOfClusters)
```

```
clt.fit(img2)
endK = time.time()

print('k means for ', noOfClusters, ' clusters took ',
      (endK - startK), ' seconds.')
retImage = clt.cluster_centers_.astype(np.uint8)[clt.labels_]

obtainedClusteredImage = remakePictureWithClusters(
    resultImage, retImage)

picReadyTime = time.time()

print('k means and cluster integration took ',
      (picReadyTime - startK), ' seconds.')


obtainedClusteredImage = cv2.cvtColor(
    obtainedClusteredImage, cv2.COLOR_RGB2BGR)

'''
centers = clt.cluster_centers_
meanList = centers.mean(axis=1)
myOrder = np.argsort(meanList)
centers = [ centers[i] for i in myOrder]
'''

#save the labels to a file for
# further reference - so that we do not need
# to rerun the K-Means to get them
labelsFolder = verifyResultsPath + '\\Labels'
if not os.path.exists(labelsFolder):
    os.makedirs(labelsFolder)
fLabels = open(labelsFolder + '\\' + file + 'Labels.csv','w')
for i in range(0, len(clt.labels_)):
    fLabels.write(str(clt.labels_[i]))
fLabels.close()
#done saving the labels in a file

#save the clusterCentersAsInitial to a file for
# further reference - so that we do not need
# to rerun the K-Means to get them
fClusters = open(labelsFolder + '\\' + file
                 + 'ClusterCenters.csv','w')
for i in range(0, len(clt.cluster_centers_)):
    for j in range(0, len(clt.cluster_centers_[0])):
        fClusters.write(
            str(clt.cluster_centers_[i][j]) + '\t')
fClusters.close()
```

```
#done saving the clusterCenters in a file

cv2.imwrite(
    folderForCurrentImage + '\\' + file + 'KMeansClass_'
    + getClass(file) + '.jpg', obtainedClusteredImage)

plt.imshow(obtainedClusteredImage)
plt.show()

# Create a colored verion of the clusters found by
# the K Means algorithm. It works up to 11 clusters
colorNames = [[0,0,255],#b
             [255,0,0],#r
             [0,128,0],#g
             [0,255,255],#aqua
             [255,153,51],#orange
             [255,255,0],#yellow
             [128,128,0], #olive
             [128,0,0],#maroon
             [255, 102, 102],#pink
             [217, 179, 140],#light chocolate
             [153, 255, 204]]#light green

colorNames = np.asarray(colorNames)
newColoredImage = colorNames[clt.labels_]
obtainedColoredImage = remakePictureWithClusters(
    resultImage, newColoredImage)

obtainedColoredImage = cv2.cvtColor(
    obtainedColoredImage, cv2.COLOR_RGB2BGR)
cv2.imwrite(
    folderForCurrentImage + '\\'
    + file + 'VeryColoredKMeansClass_'
    + getClass(file) + '.jpg', obtainedColoredImage)
#save them all in a certain folder
allFilesForThisClassFolder = (mainPath
    + 'SavedResults\\' + classInvestigated + '\\All\\')
if not os.path.exists(allFilesForThisClassFolder):
    os.makedirs(allFilesForThisClassFolder)
cv2.imwrite(
    allFilesForThisClassFolder + file
    + 'K' + str(noOfClusters)
    + 'VeryColoredKMeansClass_'
    + getClass(file) + '.jpg', obtainedColoredImage)


for i in range(0, noOfClusters):
    startPicCreation = time.time()
```

```
    newColorNames = np.ones((len(colorNames), 3))
    newColorNames[i] = colorNames[i]
    newColorNames = np.asarray(newColorNames)

    oneColoredImage = newColorNames[clt.labels_]
    obtainedOneColorImage = remakePictureWithClusters(
        resultImage, oneColoredImage)

    obtainedOneColorImage = cv2.cvtColor(
        obtainedOneColorImage, cv2.COLOR_RGB2BGR)
    if noOfClusters == 2:
        cv2.imwrite(
            folderForCurrentImage + '\\' + file
            + 'OneColoredKMeans_' + str(i)
            + '.tif', obtainedOneColorImage)
    else:
        cv2.imwrite(
            folderForCurrentImage + '\\'
            + file + 'OneColoredKMeans_'
            + str(i) + '.jpg', obtainedOneColorImage)

    endPicCreation = time.time()

    print('It took ', (endPicCreation - startPicCreation),
          ' seconds to create the picture above, that is ',
          ((endPicCreation - startPicCreation)/60),
          ' minutes.')

counter=collections.Counter(clt.labels_)

sumCounter = 0
for i in range(0, noOfClusters):
    sumCounter += counter[i]

barSizes = []
for j in range(noOfClusters):
    barSizes.append(counter[j])
getBarChart(
    clt.cluster_centers_.astype(int),
    colorNames, barSizes,
    'No of points per cluster and their colors',
    noOfClusters, folderForCurrentImage
    + '\\ClusterDistributionAndColors_' + file + '.png')
```

```
largeImage = Image.new('RGB',
                (600, noOfClusters * 100), "black")

for i in range(noOfClusters):
    # original colors
    # create image with size (300,100)
    # and background given by the cluster color

    button_img = Image.new('RGB', (300, (i+1) * 100),
            (clt.cluster_centers_.astype(np.uint8)[i][0],
             clt.cluster_centers_.astype(np.uint8)[i][1],
             clt.cluster_centers_.astype(np.uint8)[i][2]))
    if np.mean(
        clt.cluster_centers_.astype(np.uint8)[i]) > 110:
        fontColor = (0, 0, 0)
    else:
        fontColor = (255, 255, 255)
    # put text on image
    button_draw = ImageDraw.Draw(button_img)
    button_draw.text(
        (20, 70),
        str(clt.cluster_centers_.astype(np.uint8)[i])
        + ', ' + str(counter[i])
        + ' points', fill= fontColor,
        font=ImageFont.truetype("arial"))

    # put button on source image in position (0, 0)
    largeImage.paste(button_img, (0, i*100))

    #the other colors
    button_img2 = Image.new(
        'RGB', (300, (i + 1) * 100),
        (colorNames[i][0],colorNames[i][1],
        colorNames[i][2]))
    if np.mean(colorNames[i]) > 110:
        fontColor = (0, 0, 0)
    else:
        fontColor = (255, 255, 255)
    # put text on image
    button_draw2 = ImageDraw.Draw(button_img2)
    button_draw2.text((20, 70), str(colorNames[i]),
                    fill= fontColor,
                    font=ImageFont.truetype("arial"))

    # put button on source image in position (0, 0)
    largeImage.paste(button_img2, (300, i*100))

# save in new file
```

```
largeImage.save(folderForCurrentImage
                + '\\' + file
                + 'ActualClusterColors_'
                + getClass(file) + '.jpg', "JPEG")

f = open(verifyResultsPath + '\\originalClusterResults'
         + str(noOfClusters) + '.csv','a')
f.write('\n')
f.write(file + '\t' + str(clt.inertia_) + '\t' + str(w)
        + '\t' + str(h) + '\t' + str((w*h)) + '\t'
        + str(pixelsStroma) + '\t'
        + str(pixelsEpithelium) + '\t'
        + str(pixelsOthers) + '\t' + str(pixelsBackground)
        + '\t' + str(pixelsTotal) + '\t'
        + str(round((100 * pixelsStroma)/pixelsTotal, 2))
        + '\t'
        + str(round((100 * pixelsEpithelium)/pixelsTotal, 2))
        + '\t'
        + str(round((100 * pixelsOthers)/pixelsTotal, 2))
        + '\t'
        + str(round((100 * pixelsBackground)/pixelsTotal, 2))
        + '\t')
for i in range(0, len(clt.cluster_centers_)):
    for j in range(0, len(clt.cluster_centers_[0])):
        f.write(str(clt.cluster_centers_[i][j]) + '\t')

counter=collections.Counter(clt.labels_)
for i in range(0, noOfClusters):
    f.write(str(counter[i]) + '\t')
f.write(getClass(file) + '\t'
        + str(round(endK-start, 2))
        + '\t' + str(round(secondsMaskPreparation,2))
        + '\t' + str(round((endK-start)
        + secondsMaskPreparation,2)))

f.close()
print('Done with ', file + "\n**************\n")
```
